# Catalyst-Free* In Situ* Carbon Nanotube Growth in Confined Space* via* High Temperature Gradient

**DOI:** 10.1155/2018/1793784

**Published:** 2018-12-10

**Authors:** Chaoji Chen, Yanan Chen, Shuze Zhu, Jiaqi Dai, Glenn Pastel, Yonggang Yao, Dapeng Liu, Yanbin Wang, Jiayu Wan, Teng Li, Wei Luo, Liangbing Hu

**Affiliations:** ^1^Department of Materials Science and Engineering, University of Maryland, College Park, MD 20742, USA; ^2^Department of Mechanical Engineering, University of Maryland, College Park, MD 20742, USA

## Abstract

Carbonaceous materials, such as graphite, carbon nanotubes (CNTs), and graphene, are in high demand for a broad range of applications, including batteries, capacitors, and composite materials. Studies on the transformation between different types of carbon, especially from abundant and low-cost carbon to high-end carbon allotropes, have received surging interest. Here, we report that, without a catalyst or an external carbon source, biomass-derived amorphous carbon and defective reduced graphene oxide (RGO) can be quickly transformed into CNTs in highly confined spaces by high temperature Joule heating. Combined with experimental measurements and molecular dynamics simulations, we propose that Joule heating induces a high local temperature at defect sites due to the corresponding high local resistance. The resultant temperature gradient in amorphous carbon or RGO drives the migration of carbon atoms and promotes the growth of CNTs without using a catalyst or external carbon source. Our findings on the growth of CNTs in confined spaces by fast high temperature Joule heating shed light on the controlled transition between different carbon allotropes, which can be extended to the growth of other high aspect ratio nanomaterials.

## 1. Introduction

Numerous types of carbonaceous materials, such as graphite, diamond, carbon fiber, and activated carbon, have been extensively investigated and commercialized for various applications, such as batteries, supercapacitors, and composite materials [1–4]. Recently, new carbon allotropes, including fullerene [5–7], carbon nanotubes (CNTs) [8–13], and grapheme [14–17], have been discovered, which offer attractive physicochemical properties. However, to date, production of these and other high-end carbon allotropes remains expensive. Typically, catalysts and external carbon sources are used to promote the scalable synthesis and preferential nucleation of low-dimensional carbon allotropes [18–22]. Given their identical chemical composition, the direct transformation of abundant and low-cost allotropes, such as amorphous carbon and graphite, to high-end CNTs and graphene, is highly desirable for commercial and industrial applications.

Many techniques have been established to transition between carbon allotropes. One of the most prototypical transformations is the preparation of C_60_ from graphite using laser ablation, electron beam evaporation, or arc-discharge methods [23]. During the arc-discharge method, graphite is sublimated at extremely high temperatures and then carbon molecules reform into C_60_. Another valuable allotropic transition is the production of expensive diamond from low-cost carbon allotropes from high temperature/pressure conditions or chemical vapor deposition [24–26]. Although transitions between various carbon allotropes have been extensively studied, there has yet to be a scalable method for the transformation of amorphous carbon or graphite to CNTs without the use of a catalyst.

**Figure 1:**
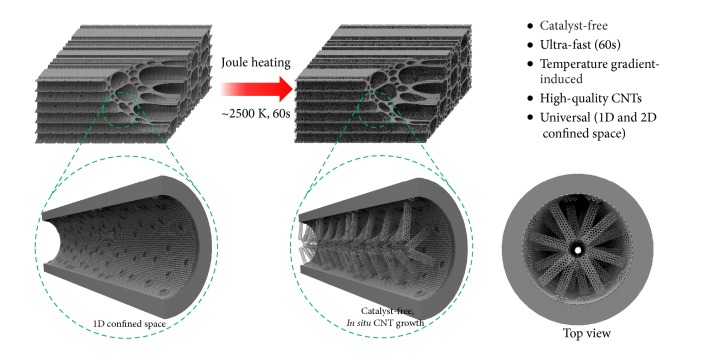
**Schematic of the catalyst-free,* in situ* CNT formation method in the long 1D confined channels of C-wood by Joule heating without the need for an external carbon source.** Numerous multiwall CNTs were grown in the channels (i.e., vessels and lumina) of C-wood after 1 min of Joule heating at ~2500 K.

Herein, we report a CNT synthesis method based on biomass-derived materials without the use of a catalyst or external carbon source. Rapid and scalable Joule heating can effectively generate ultrahigh temperatures compared to traditional heating processes (e.g., thermal radiation using an electric furnace), and in this manner we were able to transform abundant and low-cost wood-derived amorphous carbon and defective reduced graphene oxide (RGO) into CNTs in just 1 min without any catalyst or additional carbon source (Figure 1). Additionally, due to the existence of defective carbon sites or structures in carbonized wood (C-wood) and RGO, a temperature gradient occurs during Joule heating, which molecular dynamics (MD) simulations suggest plays a critical role in the migration of carbon atoms and the formation of CNTs. The confined environment is also important for CNT synthesis, as evidenced in the one-dimensional (1D) long channels of carbonized wood and layer-layer spacing of 2D RGO nanosheets. Due to the abundance and low cost of wood and graphite, this rapid and simple method can improve CNT production and other allotrope transitions for commercial applications.

## 2. Results

### 2.1. Morphology and Microstructure of Carbonized Wood

**Figure 2:**
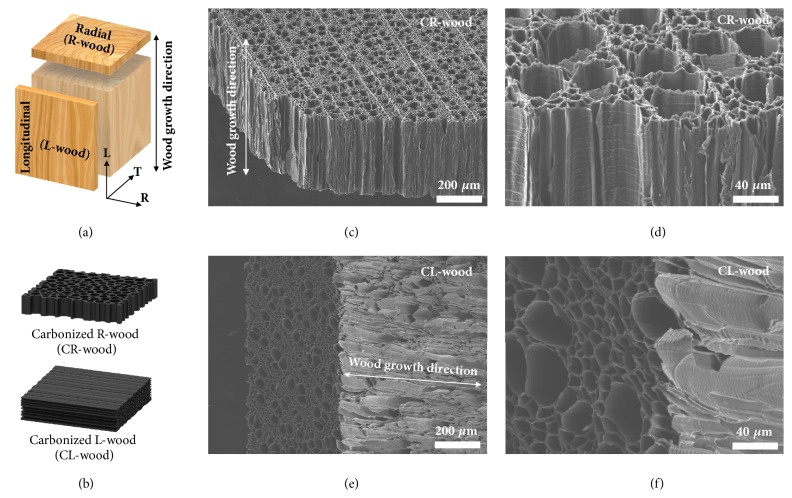
**Morphology and microstructure of the CR-wood and CL-wood.** (a, b) Schematic indicating the two types of wood pieces obtained by slicing wood bricks along the radial or longitudinal directions before and after carbonization at 1000°C. (c-f) SEM images of CR-wood (c, d) and CL-wood (e, f) prove that the vessel structures are well maintained after the carbonization process.

Wood exhibits anisotropic properties based on its growth direction [27–30]. In this work, we studied different orientations of wood for the CNT growth process. R-wood was obtained by radially slicing a wood brick (i.e., perpendicular to the growth direction), while a longitudinal slice was denoted as L-wood (Figure 2(a)). Typically, a large number of long and well-aligned vessels exist along the growth direction in wood for transporting water and minerals from the roots up to the leaves and other parts of the tree [31, 32]. Thus, numerous vessels were observed in both R-wood and L-wood with contrasting orientations ([Sec supplementary-material-1]). The wood pieces were carbonized at 1000°C under an argon atmosphere to yield carbonized radial wood (CR-wood) and carbonized longitudinal wood (CL-wood) (Figure 2(b)). The vessel structures in wood are well maintained after carbonization, as confirmed by scanning electron microscopy (SEM) images (Figures 2(c)–2(f)). X-ray diffraction (XRD) patterns in [Sec supplementary-material-1]a indicate the amorphous carbon nature of CR-wood and CL-wood, where two broad peaks at about 24° and 43° can be indexed to the (002) and (101) planes. This is in good agreement with other biomass-derived carbon materials obtained by carbonization at temperatures near 1000°C [33–35]. [Sec supplementary-material-1]b exhibits the Raman spectra of CR-wood and CL-wood, which present two bands at 1330 cm^−1^ (D-band) and 1595 cm^−1^ (G-band), indicative of sp^3^ and sp^2^ hybridized carbon atoms, respectively. The intensity ratio of the D-band over the G-band (I_*D*_/I_*G*_) is about 1.2, which like the XRD results indicates the amorphous structures of the materials. The high-resolution transmission electron microscopy (HRTEM) image in [Sec supplementary-material-1] further confirms the amorphous structure of the samples, with small fragments of graphene layers.

### 2.2. External Catalyst- and Carbon Source-Free Synthesis of CNTs in 1D Confined Space

**Figure 3:**
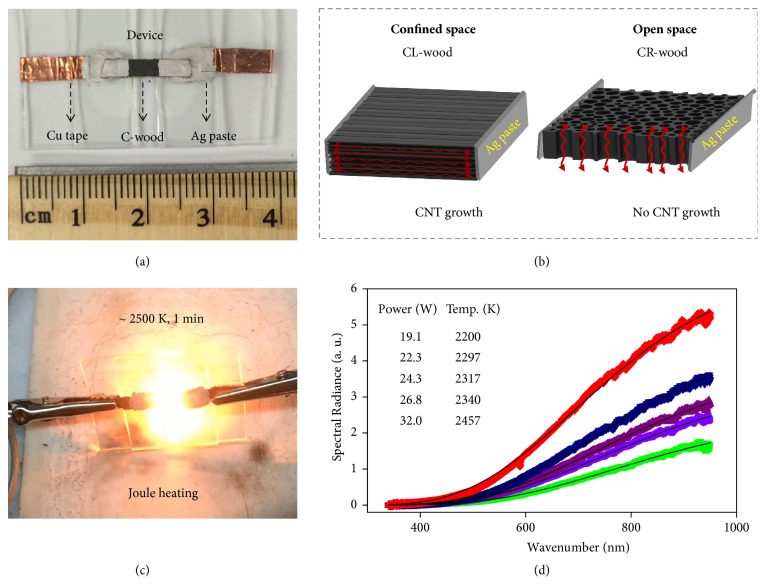
**High temperature Joule heating of wood-derived amorphous carbon.** (a) Configuration of the Joule heating process. (b) For CL-wood, the vessels are long, and the ends of the sample are sealed by silver paste to form a 1D confined space; for CR-wood, the vessels were short and open to the argon atmosphere. (c) An image depicting the bright light emitted during the Joule heating process of C-wood. (d) Temperature measurements at different powers using a spectrometer coupled with optical fiber.

To conduct the high temperature Joule heating process, a piece of carbonized wood with dimensions of 5 mm × 3 mm and a thickness of 500 *μ*m was bridged between two glass slides and contacted by silver paste on copper tape (Figure 3(a)). As depicted in Figure 3(b), for the CL-wood, the vessels along the length direction are long and form a 1D confined space when the vessels are sealed by silver paste at the two ends. For the CR-wood specimen, the vessels were short, normal to the cross-section, and open to the environment. The as-assembled devices were transferred into an Ar-filled glove box for Joule heating. A direct current (DC) power input was applied so that the carbonized wood exhibited bright light, indicating a high temperature in the sample (Figure 3(c)). After loading with a power of ~32 W for 1 min, the DC input was removed, and the wood samples were collected. To more precisely identify the temperature of the Joule heated carbonized wood, we collected the emitted radiation spectra from 350 nm to 950 nm using an optical fiber (400 *μ*m diameter, ocean optics) and fitted the spectra to gray body radiation curves (Figure 3(d)). The temperature (T) of the carbonized wood during Joule heating can be estimated according to Planck's distribution function using a constant (“gray”) emissivity *ε*_*gray*_:

(1) $$\begin{array}{c} B_{\lambda }\left(\;\lambda ,T\;\right)=\gamma \varepsilon_{gray}\frac{2hc^{2}}{\lambda^{5}}\frac{1}{e^{hc/\lambda k_{B}T}-1}, \end{array}$$

in which* h* is Planck's constant,* c* is the speed of light, *λ* is the wavelength, *k*_B_ is the Boltzmann constant, and the scaling constant *γ* is introduced for fitting. According to the spectra, the highest temperatures of the CL-wood and CR-wood during Joule heating at 32 W were approximately the same, 2457 K ± 50 K [36–39].

**Figure 4:**
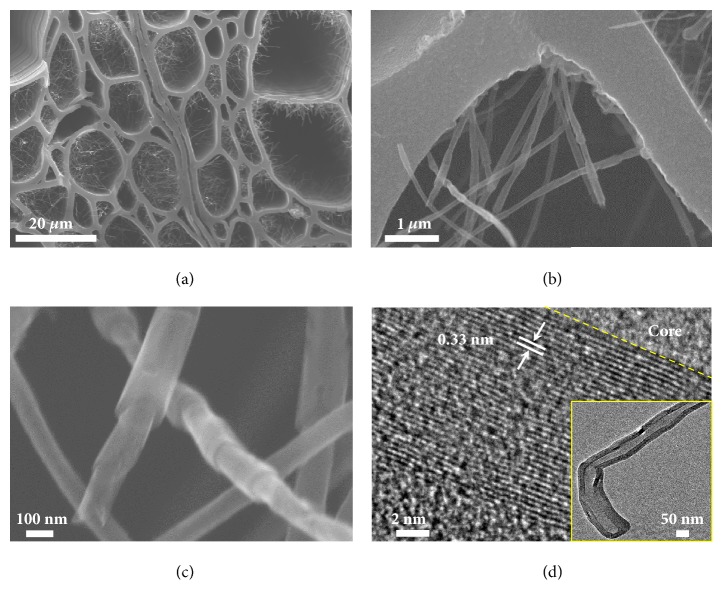
**Morphology observations of CNT growth in the channels of CL-wood after 1 min Joule heating. **(a, b) Low-magnification SEM images of CL-wood after Joule heating showing numerous stacked cup-structured CNTs grew in the vessels. (c) High-magnification SEM image of CNTs, indicating the “stacked cup” structure. (d) HRTEM and TEM (inset) images of CNTs, confirming the hollow core, highly crystalline structure of the inner wall and amorphous structure of the outer wall.

SEM imaging shows that the overall morphology and structure of the carbonized wood is well maintained after the Joule heating process ([Sec supplementary-material-1] and [Sec supplementary-material-1]). When magnifying the CL-wood vessels, we observed large quantities of CNTs within the channels (Figures 4(a) and 4(b)). The SEM images show that the length of these CNTs can reach 20 *μ*m, while the diameters range from 100 nm to 160 nm. More interestingly, these CNTs have a unique “stacked cup” structure and grow from the vessel wall with many joints (Figure 4(c) and [Sec supplementary-material-1]), which is similar to the previously reported vapor grown carbon nanofibers at a high temperature of 2800°C [40]. TEM and HRTEM images were taken to provide further insight. The inset in Figure 4(d) confirms the hollow structure of a typical CNT with a diameter of ~100 nm. HRTEM images also reveal the highly crystalline structure of the inner wall of the nanotubes with a d-spacing of 0.33 nm (Figure 4(d)), which is assigned to the interplanar spacing between the (002) planes of graphitic carbon. It is noteworthy that the outer wall of the nanotubes is amorphous (Figure 4(d) and [Sec supplementary-material-1]), which is significantly different from CNTs grown by other methods. We ascribe the amorphous structure of the outer wall to the unique temperature gradient-induced formation process of the CNT in the 1D confined space. Upon Joule heating, crystalline carbon* in situ* grows under a high temperature gradient, while during the cooling process, the temperature decreases and less crystalline carbon deposits on the outer surface of the as-grown CNT, forming an amorphous layer on the outer wall. We also notice that there were no CNTs observed in the CR-wood vessels after the same Joule heating process due to its open structure without space confinement ([Sec supplementary-material-1]). In addition, the CL-wood failed to grow CNT at a lower Joule heating temperature (e.g., 1500 K) owning to the relatively low temperature gradient. We anticipate that the as-synthesized carbonized wood-CNT composites may have a variety of potential applications in energy storage (e.g., supercapacitors, lithium-sulfur batteries, sodium-ion batteries, and flow batteries), electrocatalyst and photocatalyst, water treatment (e.g., solar desalination, heavy metal removal, and oil absorption), and microwave absorption by taking advantage of their unique multichanneled structure and CNT network.

### 2.3. Formation Mechanism of CNTs in 1D Confined Space

**Figure 5:**
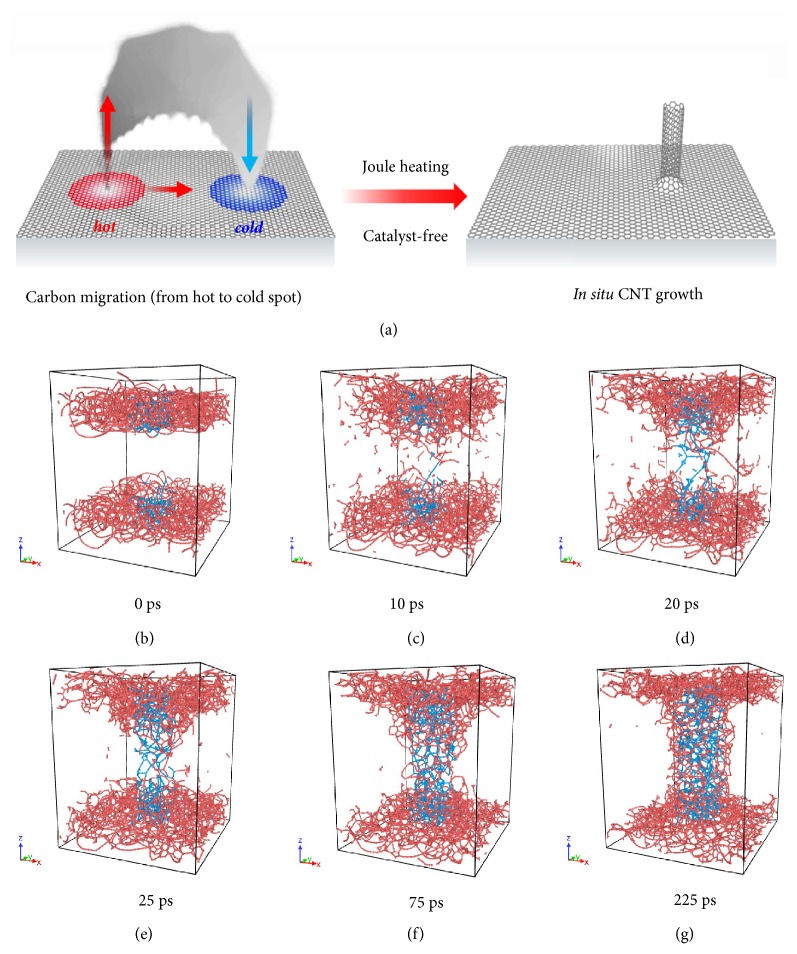
**MD simulations of the catalyst-free,* in situ*, and fast formation of CNTs within the confined carbon structure.** (a) Schematic showing the proposed mechanism of CNT growth in the confined carbon structure by Joule heating. The defect sites on C-wood have a higher temperature during Joule heating, which promotes the migration of the carbon atoms from high temperature regions (red) to low temperature regions (blue) and results in CNT growth. (b-g) MD simulation results verify the 1D CNT structure formation in the confined space* via* a substantial temperature gradient. The blue atoms belong to the low temperature region (300 K) while the red atoms belong to the high temperature region (3000 K).

To reveal the formation mechanism of CNTs in the CL-wood vessels without contributions from a catalyst or external carbon source, we hypothesized that the reaction process proceeds as illustrated in Figure 5(a). Due to the low temperature carbonization process (1000°C), a large number of defect sites exist in C-wood. When the C-wood is subjected to Joule heating, defect sites with higher resistance generate higher local temperatures than regions without defects, leading to temperature gradient [41]. The temperature gradient can cause carbon atoms to migrate from regions with higher local temperature towards regions with lower local temperature (Figure 5(a)). On the other hand, carbon atoms located at defect sites are involved in the carbon sublimation process due to the unstable and high temperature environment. When CL-wood was used, these carbon atoms can be confined in the long, 1D confined vessels. As a result, these atoms continue to grow as CNTs. In contrast, when there is no confined space, as in the CR-wood, carbon atoms can readily escape from the open ends, thus CNTs are not formed during the Joule heating process.

To further confirm this carbon transformation mechanism, we carried out MD simulations (see more simulation details in the Supporting Information and [Sec supplementary-material-1]), in which we used the large-scale atomic/molecular massively parallel simulator (LAMMPS) [42] and the REAXFF potential [42, 43]. We designed the cylindrical volume perpendicular to the amorphous carbon vessel wall inside the simulation box as the low temperature region, and everywhere else was made the high temperature region. In order to highlight the role of the temperature gradient in an expedited migration process, 3000 K was assigned to the high temperature region and 300 K to the low temperature region. Figures 5(b)–5(g) shows the evolution of configuration, in which a typical 1D CNT structure was formed.

### 2.4. Extending the External Catalyst- and Carbon Source-Free Synthesis of CNTs from 1D to 2D Confined Space

**Figure 6:**
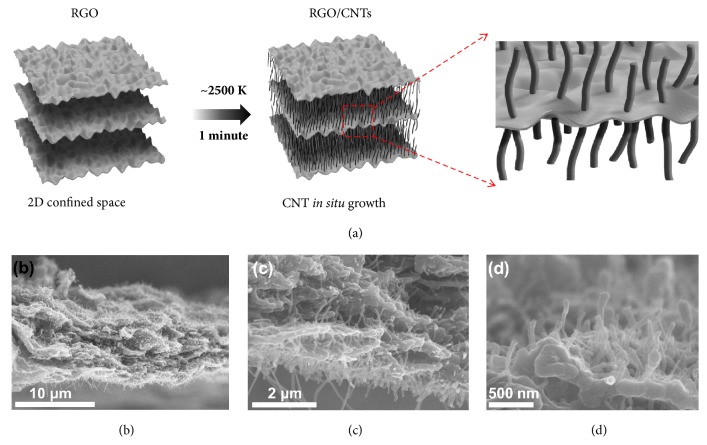
**Growth of CNTs in between confined RGO layers using Joule heating without a catalyst or external carbon source. **(a) A schematic showing the growth of CNTs in between the RGO layers. (b-d) Low- and high-magnification SEM images of CNTs in between RGO layers.

We can extend the CNT growth from wood with 1D long/closed vessels to two-dimensional (2D) materials, with the additional condition that the 2D nanosheets are relatively impenetrable to gas species [44, 45]. To test this proposed growth mechanism in between confined carbon layers, we made RGO using a modified Hummers' method [46] (Figure 6(a)). After 1 min Joule heating, the surface of the RGO sample was carefully peeled off and characterized by SEM. As shown in Figures 6(b)–6(d), it is clear that numerous CNTs are present in between the RGO layers, even without the use of any catalysts or external carbon sources. It is well known that RGO made by Hummers' method with low temperature reduction has abundant defect sites [47, 48]. We believe that a temperature gradient occurs on the RGO nanosheets during the Joule heating process and leads to carbon atom migration and CNT formation, in a similar fashion as in CL-wood. In comparison to CNTs obtained in vessels of C-wood, CNTs grown in between RGO layers are shorter, which can be attributed to the limited space in between the 2D RGO nanosheets relative to the long 1D vessels of the CL-wood.

## 3. Discussion

In brief, we have demonstrated for the first time that CNTs can be directly grown in both 1D confined channels of carbonized wood and 2D confined spaces of RGO by a fast Joule heating process without any catalysts. In contrast to other CNT growth methods, no external carbon source is needed because the confined materials (wood-based carbon or RGO) are laden with defects and serve as ideal carbon sources. MD simulations show that transformation from amorphous carbon and RGO to CNTs depends on the temperature gradient, which results from the high resistance of defect sites during Joule heating. Carbon atoms migrate from high temperature regions towards lower temperature regions and grow into CNTs in confined spaces. Further analysis can shed light on the transformation mechanisms between carbon allotropes. Our findings hold promise for the growth of a range of high aspect ratio nanomaterials, such as metal nanowires and other nanostructures, at high temperatures in confined spaces.

## 4. Materials and Methods

### 4.1. Preparation of Carbonized Wood and RGO Film

In this study, wood blocks (American basswood) were and purchased from Walnut hollow Inc. and used as the starting material. The wood block was cut in two directions (radial and longitudinal, as schematically shown in Figure 2(a)). The obtained wood slices were then transferred into a tube furnace and heated at 260°C for 8 h in air followed by carbonization at 1000°C under argon for 2 h.

A graphene oxide (GO) dispersion was prepared using a modified Hummer method [1]. Freestanding GO films were obtained by vacuum filtration using the as-prepared GO dispersion. Before Joule heating, freestanding GO films were reduced in a tube furnace at 500°C in argon for 30 min to produce the freestanding reduced graphene oxide (RGO) films.

### 4.2. Joule Heating Treatment

To conduct the Joule heating treatment, the setup shown in Figure 3(a) was used, in which carbonized wood or freestanding RGO film was bridged between two glass slides and contacted by silver paste on copper tape. The setup was then transferred into an argon-filled glove box. A direct current (DC) power input was applied using a power transformer (Volteq HY6020EX). The emitted light during Joule heating was collected using an optical fiber (400 *μ*m diameter) and connected to a spectrometer (ocean optics). The measurement system was calibrated by a National Institute of Standards and Technology (NIST)-traceable light source.

### 4.3. Characterization

XRD patterns were collected using a D8 Advanced (Bruker AXS, WI, USA). Raman spectra were recorded by a Horiba Jobin-Yvon system with a laser wavelength of 532 nm. The morphology of the samples was examined by SEM (Hitachi SU-70 field emission scanning electron microscopy) and TEM (JEM 2100 FE-TEM).

## Supplementary Materials

Supplementary Material
** Figure S1: **SEM images of (a) R-wood and (b) L-wood. A large number of long and nearly aligned channels (vessels and lumina) exist in wood along the tree growth direction for transporting water and minerals, which are well maintained after carbonization at 1000°C under argon.** Figure S2: **(a) XRD patterns of wood and carbonized wood (C-wood). (b) Raman spectra of CR-wood and CL-wood. Due to the low carbonization temperature, CR-wood and CL-wood exhibit an amorphous structure, where defect sites will generate high temperature during Joule heating due to their high resistance.** Figure S3: **HRTEM images of (a) CR-wood and (b) CL-wood, indicating the amorphous structure. During Joule heating, defective carbon serves as the carbon source for high temperature carbon transformation.** Figure S4: **morphology observations of CR-wood after 1 min Joule heating. (a) Cross-sectional and (b) top view SEM images. (c, d) Magnified SEM images of (a). The overall morphology of the CR-wood was maintained after the Joule heating process.** Figure S5: **morphology observations of CL-wood after 1 min Joule heating. (a) Top view and (b) cross-sectional view SEM images. The overall morphology of CL-wood was also maintained after Joule heating, while CNTs were observed in the channels under high-resolution SEM (Figure 4).** Figure S6: **(a) TEM and (b) HRTEM images of the joint of the “stacked cup” structured CNT grown in the confined wood channel.** Figure S7: **HRTEM image of the as-synthesized CNT in the confined wood channel with crystalline inner wall and amorphous outer wall.** Figure S8: **high-magnification SEM image of CR-wood after 1 min Joule heating, in which no CNTs were observed.** Figure S9:** simulation model. The dashed blue lines indicate the simulation box. The red cylinder indicates the volume in the simulation box that was subjected to a relatively low temperature, while the other volume in the simulation box was subjected to a high temperature.
