# Supplementary material for: Catalyst-Free In Situ Carbon Nanotube Growth in Confined Space via High Temperature Gradient
Source: Research (Wash D C). 2018 Dec 10;2018:1793784. doi: 10.1155/2018/1793784 (PMC6750109; doi:10.1155/2018/1793784)
Supplement: Supplementary Material — Figure S1: SEM images of (a) R-wood and (b) L-wood. A large number of long and nearly aligned channels (vessels and lumina) exist in wood along the tree growth direction for transporting water and minerals, which are well maintained after carbonization at 1000°C under argon. Figure S2: (a) XRD patterns of wood and carbonized wood (C-wood). (b) Raman spectra of CR-wood and CL-wood. Due to the low carbonization temperature, CR-wood and CL-wood exhibit an amorphous structure, where defect sites will generate high temperature during Joule heating due to their high resistance. Figure S3: HRTEM images of (a) CR-wood and (b) CL-wood, indicating the amorphous structure. During Joule heating, defective carbon serves as the carbon source for high temperature carbon transformation. Figure S4: morphology observations of CR-wood after 1 min Joule heating. (a) Cross-sectional and (b) top view SEM images. (c, d) Magnified SEM images of (a). The overall morphology of the CR-wood was maintained after the Joule heating process. Figure S5: morphology observations of CL-wood after 1 min Joule heating. (a) Top view and (b) cross-sectional view SEM images. The overall morphology of CL-wood was also maintained after Joule heating, while CNTs were observed in the channels under high-resolution SEM (Figure 4). Figure S6: (a) TEM and (b) HRTEM images of the joint of the “stacked cup” structured CNT grown in the confined wood channel. Figure S7: HRTEM image of the as-synthesized CNT in the confined wood channel with crystalline inner wall and amorphous outer wall. Figure S8: high-magnification SEM image of CR-wood after 1 min Joule heating, in which no CNTs were observed. Figure S9: simulation model. The dashed blue lines indicate the simulation box. The red cylinder indicates the volume in the simulation box that was subjected to a relatively low temperature, while the other volume in the simulation box was subjected to a high temperature. [file 1793784.f1.docx]

**Supporting information**

**Catalyst-Free *in situ* Carbon Nanotube Growth in Confined Space *via* High Temperature Gradient**

Chaoji Chen,^1‡^ Yanan Chen,^1‡^ Shuze Zhu,^2‡^ Jiaqi Dai,^1^ Glenn Pastel,^1^ Yonggang Yao,^1^ Dapeng Liu,^1^ Yanbin Wang,^1,2^ Jiayu Wan,^1^ Teng Li,^2^ Wei Luo,^1,2,3^* Liangbing Hu^1^*

^1^Department of Materials Science and Engineering, University of Maryland, College Park, Maryland, 20742

^2^Department of Mechanical Engineering, University of Maryland, College Park, Maryland, 20742

^3^Current address: School of Materials Science and Engineering, Tongji University, Shanghai, China, 201804

^‡^These authors contribute equally

*Email: [binghu@umd.edu](mailto:binghu@umd.edu); weiluo@tongji.edu.cn


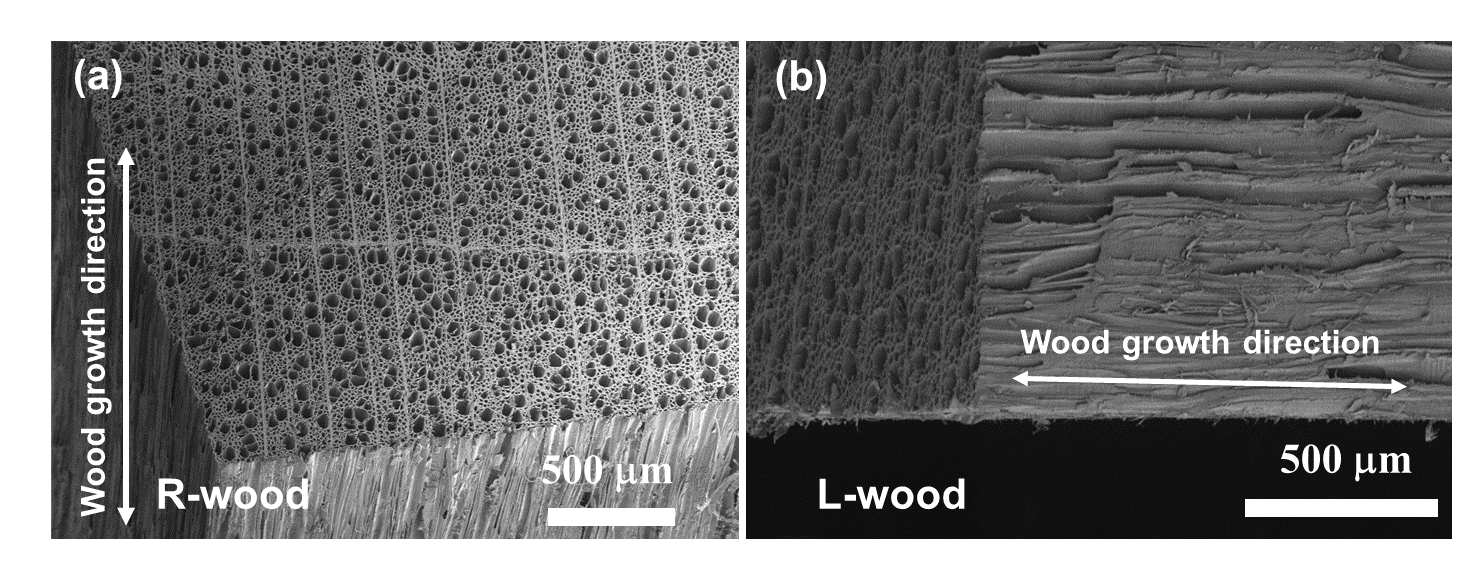


**Figure S1.** SEM images of (a) R-wood and (b) L-wood. A large number of long and nearly aligned channels (vessels and lumina) exist in wood along the tree growth direction for transporting water and minerals, which are well-maintained after carbonization at 1000 °C under argon.


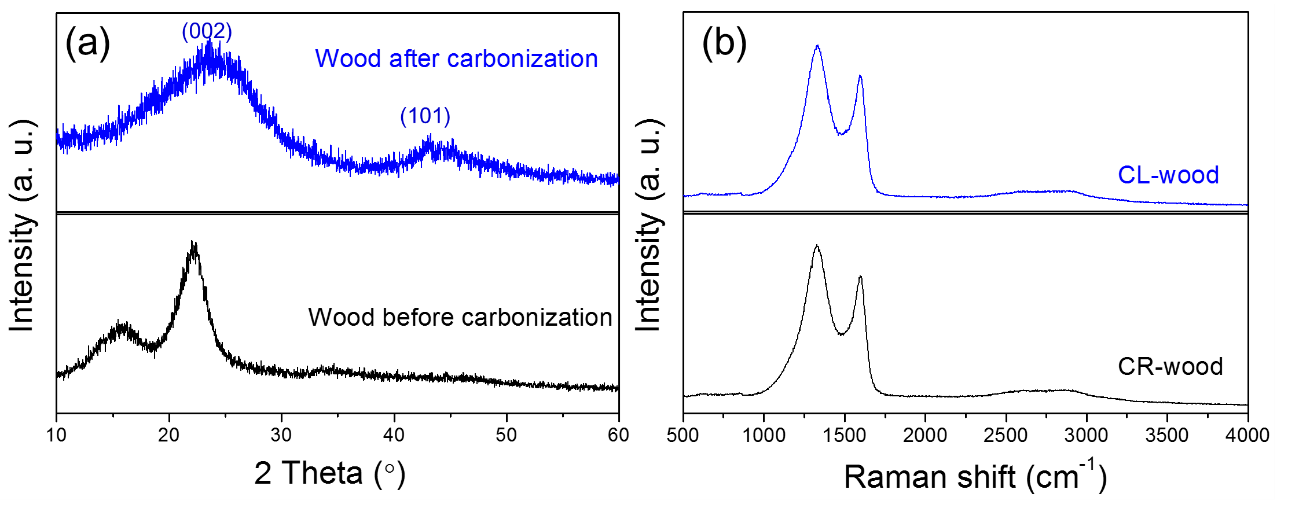


**Figure S2.** (a) XRD patterns of wood and carbonized wood (C-wood). (b) Raman spectra of CR-wood and CL-wood. Due to the low carbonization temperature, CR-wood and CL-wood exhibit an amorphous structure, where defect sites will generate high temperature during Joule heating due to their high resistance.


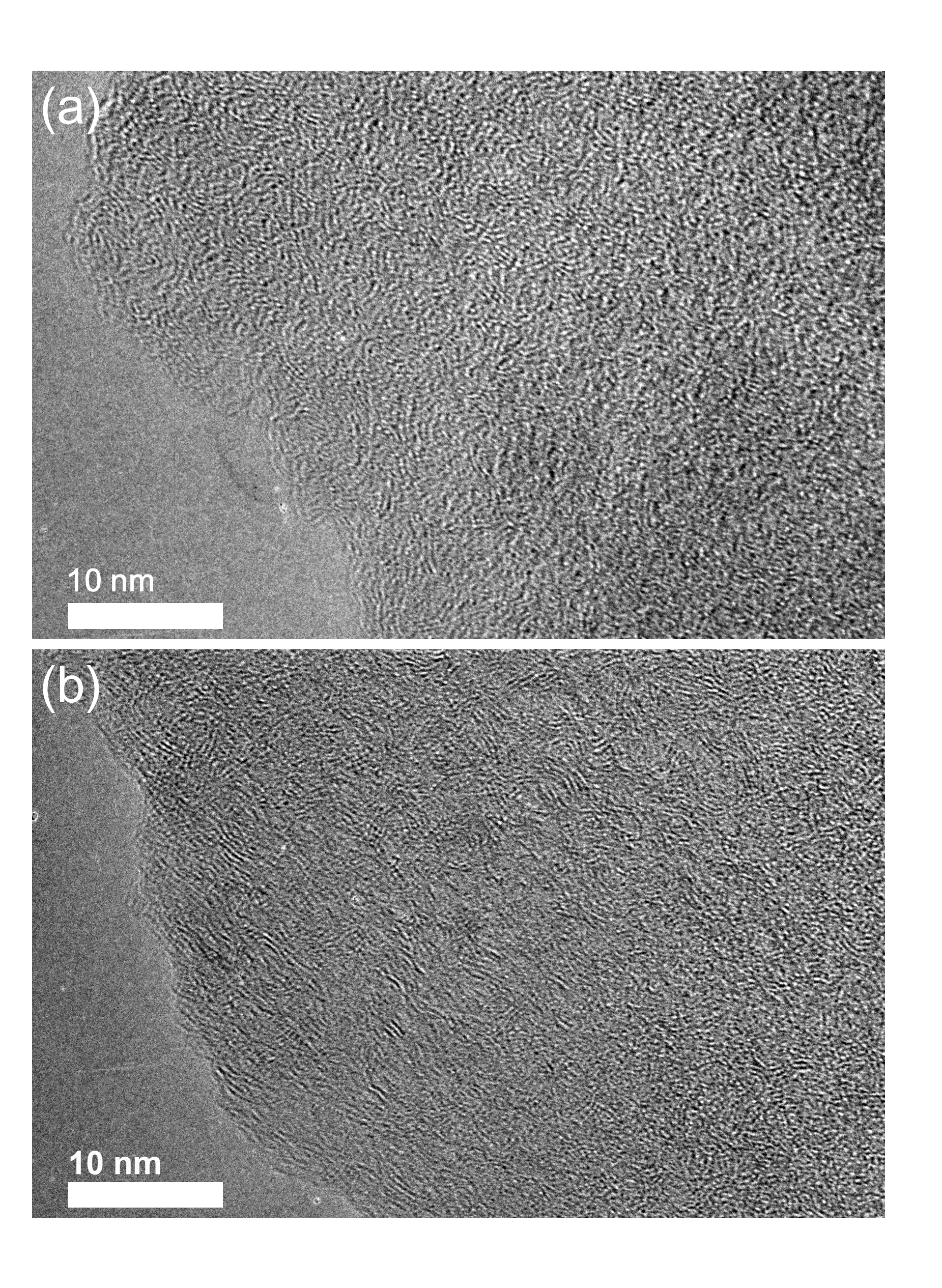


**Figure S3.** HRTEM images of (a) CR-wood and (b) CL-wood, indicating the amorphous structure. During Joule heating, defective carbon serves as the carbon source for high temperature carbon transformation.

**
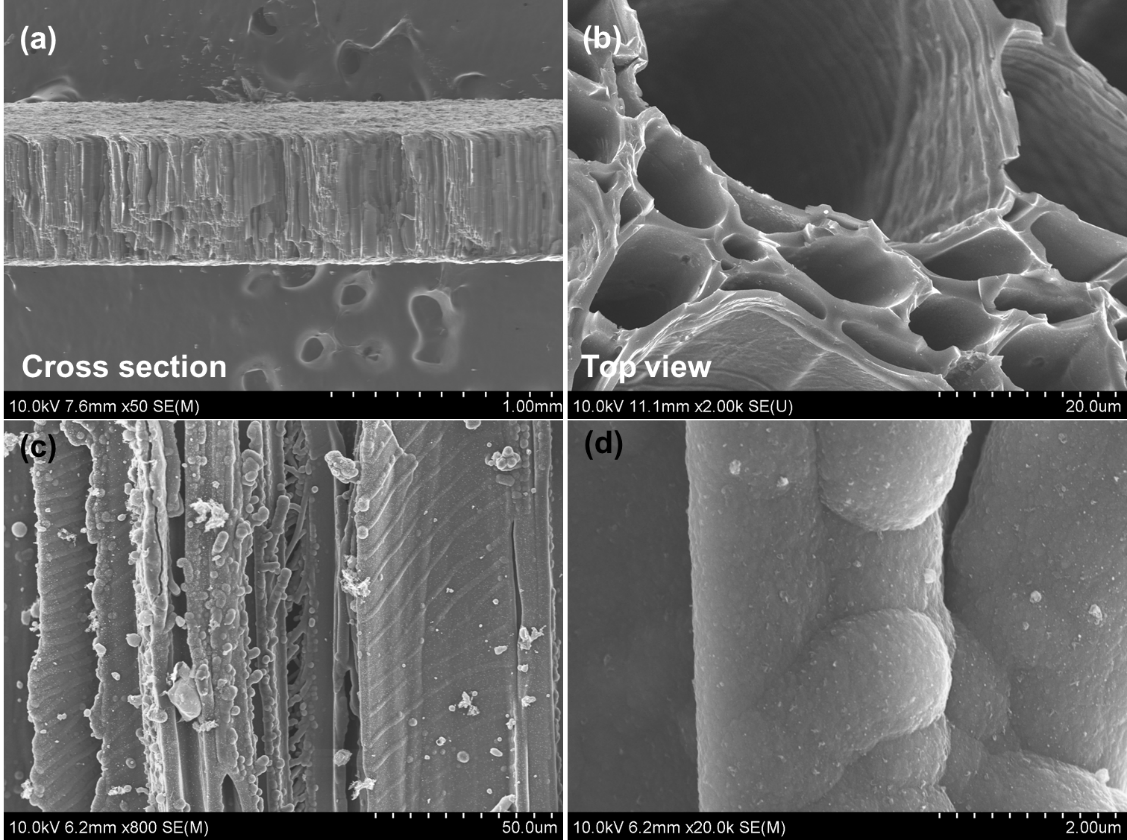
**

**Figure S4.** Morphology observations of CR-wood after 1 min Joule heating. (a) Cross-sectional and (b) top view SEM images. (c, d) Magnified SEM images of (a). The overall morphology of the CR-wood was maintained after the Joule heating process.

**
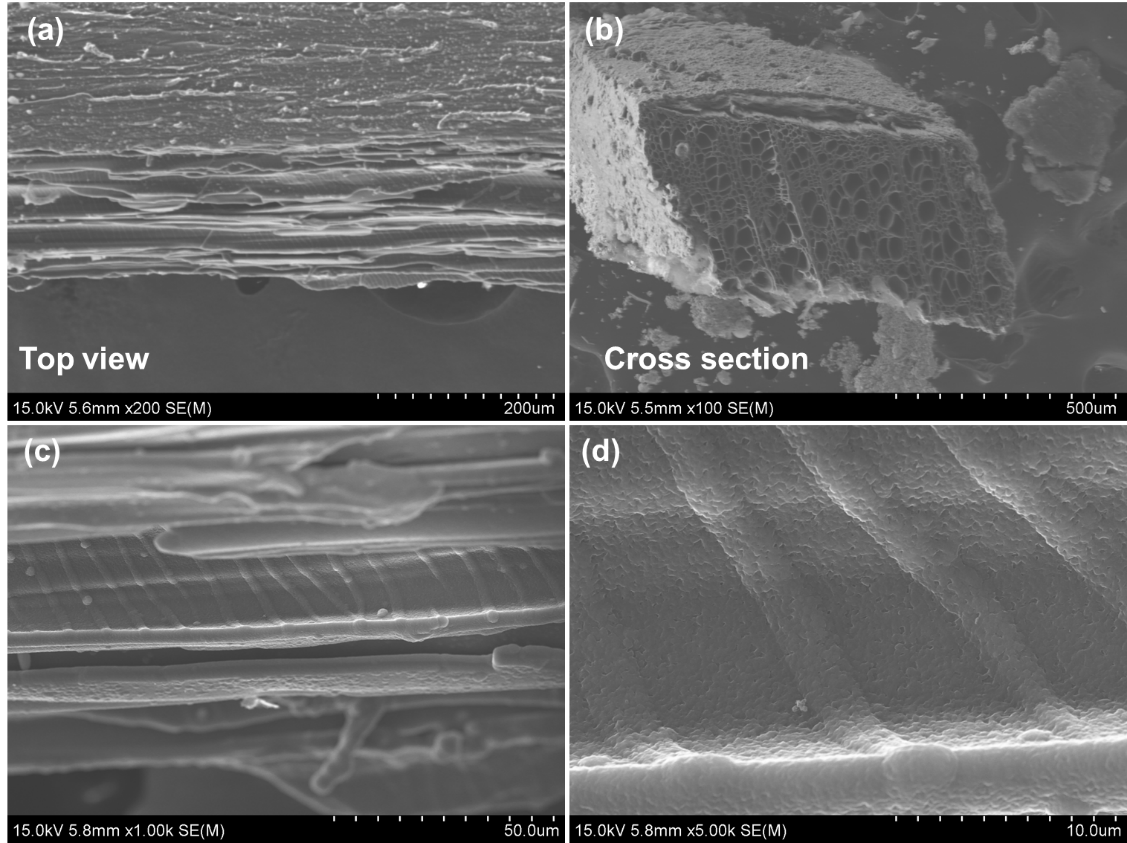
**

**Figure S5.** Morphology observations of CL-wood after 1 min Joule heating. (a) Top view and (b) cross-sectional view SEM images. The overall morphology of CL-wood was also maintained after Joule heating, while CNTs were observed in the channels under high-resolution SEM (Figure 4).

**
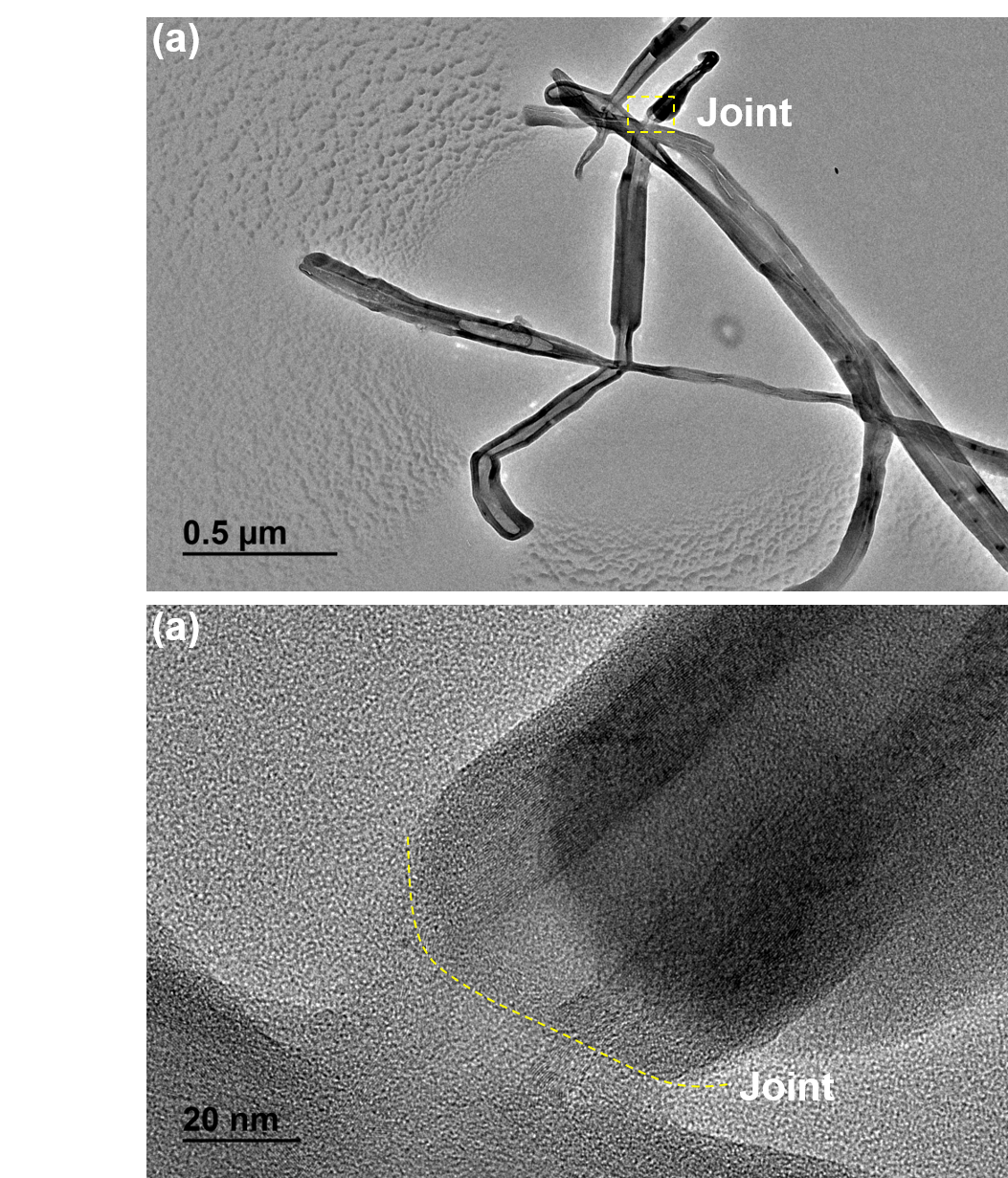
**

**Figure S6.** (a) TEM and (b) HRTEM images of the joint of the “stacked cup” structured CNT grown in the confined wood channel.


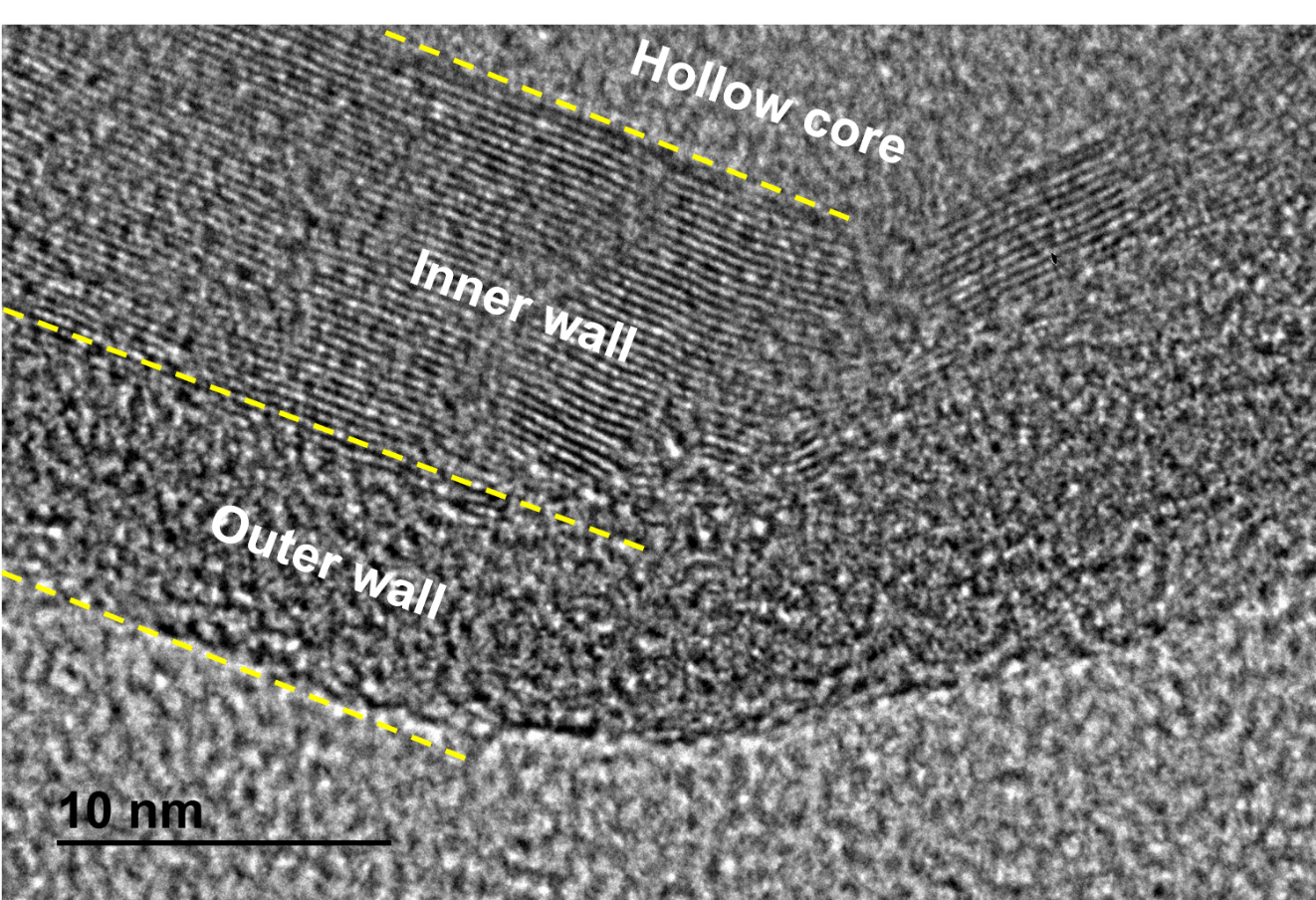


**Figure S7.** HRTEM image of the as-synthesized CNT in the confined wood channel with crystalline inner wall and amorphous outer wall.

**
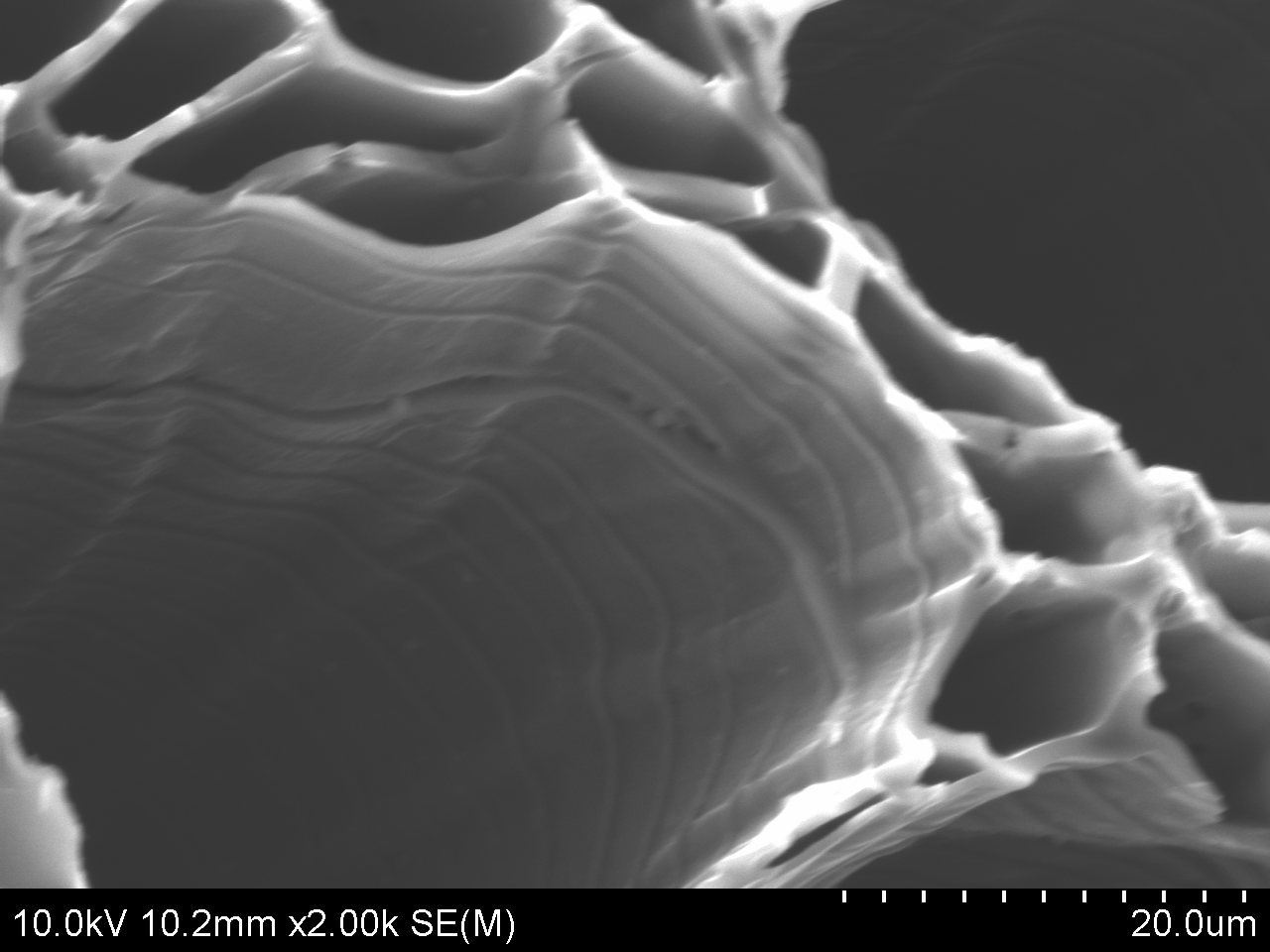
**

**Figure S8.** High-magnification SEM image of CR-wood after 1 min Joule heating, in which no CNTs were observed.


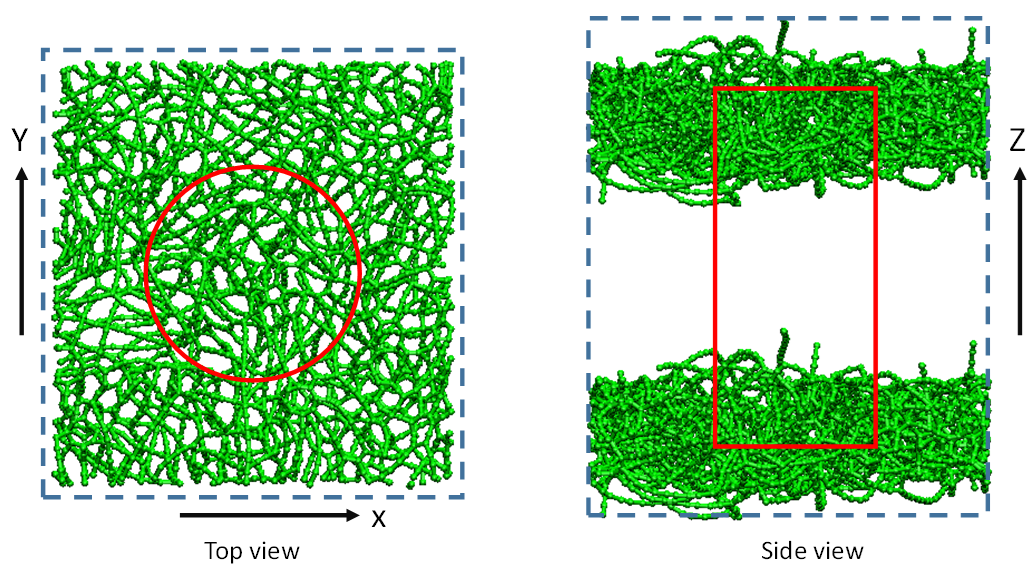


**Figure S9.** Simulation model. The dashed blue lines indicate the simulation box. The red cylinder indicates the volume in the simulation box that was subjected to a relatively low temperature, while the other volume in the simulation box was subjected to a high temperature.

The time step was set to 0.25 fs. The simulation was performed on a canonical ensemble (NVT), controlled by a Nosé-Hoover thermostat. The red cylinder in Figure S9 indicates the volume in the simulation box that was subjected to a relatively low temperature (300 K), while the other volume in the simulation box was subjected to 3000 K. The carbon atoms that were included in this cylindrical volume were defined cyclically every 5000 time steps. Periodical boundary conditions were imposed along the X and Y directions, while a reflective wall condition was applied at the Z boundary.

**References**

1, D. C. Marcano, D. V. Kosynkin, J. M. Berlin, A. Sinitskii, Z. Sun, A. Slesarev, L. B. Alemany, W. Lu and J. M. Tour, ***ACS Nano***, 2010, *4*, 4806-4814.
